# Developing energy based benchmark model and detailed energy analysis through school statistical data and field surveys

**DOI:** 10.1016/j.heliyon.2022.e10958

**Published:** 2022-10-05

**Authors:** Beungyong Park, Byeong-Un Kang, Doo-Yong Park

**Affiliations:** a Department of Building and Plant Engineering, Hanbat National University, Daejeon 34158, South Korea; b Research Institute, WooWon M&E Inc, Seoul 08768, South Korea; c Energy Division, Korea Conformity Laboratories (KCL), Seoul 06711, South Korea

**Keywords:** School building, Energy based benchmark model, School information, Energy consumption, Energy saving

## Abstract

Approximately 20% of all school facilities, i.e., 7,980 buildings, are at least 40 years old in Korea. As the number of aged buildings is expected to rapidly increase, it is necessary to improve such facilities to protect safe learning environments and save energy. This study aims to develop the energy based benchmark model (EBBM) and utilization for energy saving in school. EBBM can be operated for school buildings, a category encompassing complex buildings with different systems and large gaps between them. To utilization of EBBM allows us to obtain knowledge from the school facility, in order to define and tune data driven analysis rules. Data driven energy analysis also allows ascertaining their expected energy consumption and estimating the possible saving systems by using the building energy flow chart. In this research, Korea education data-base was analyzed to classify energy based benchmark model. Applicability review is conducted through detailed system analysis of 10 schools. The difference between the benchmark model calculated value and the actual value was confirmed to be within 10%. Through the benchmarking model, it is possible to compare energy consumption for buildings of different sizes. Energy consumption by energy source and room type was analyzed through an in-depth status survey. Most of School Classrooms consume the largest amount of energy, with cooling and heating energy constituting the largest proportion.

## 1 Introduction

New obligations worldwide to cut greenhouse gas emissions and conserver energy. The energy consumption was increasing in building sector, such as houses and schools. Approximately 20% of all school facilities, i.e., 7,980 buildings, are at least 40 years old in Korea. As the number of aged buildings is expected to increase rapidly it is urgently necessary to improve such facilities to protect safe learning environments and save energy in Korea. The Korea Ministry of Education decided to turn 2,835 old school buildings aged 40 years and over into green smart schools as the first phase of the project by investing 15.4 billion dollars for 5 years from 2021 to 2025. The details of the green smart school include the green schools oriented towards low-carbon zero energy [1].

Energy management for schools has become a concern and for this purpose, schools must have standard energy requirements and ensure a high level of indoor environmental comfort. It is to improve energy through the improvement of educational environment facilities and the remodeling of old educational facilities. In order to save energy in school facilities, it is necessary to understand the factors of energy saving in terms of users.

Previous studies related to energy in educational facilities have mostly focused on the performance evaluation of remodeling alternatives through simulation and the analysis of the energy use status by analyzing the statistical data of educational facilities.

Lee et al. analyzed the energy-saving effect of each remodeling element technology for reducing energy consumption in old high school buildings through simulation using the Visual DOE 4.0 software. The analyzed element technologies were mainly limited to the insulation, natural lighting, control (system efficiency and indoor set temperature), and window sectors [2]. Yoon et al. analyzed the energy use status of middle school facilities nationwide by region and energy source using the educational statistics data service. They also analyzed the phenomenon and cause of energy consumption. When various energy sources were converted into one unit and combined, the proportion of each energy source was found to be 70.2% for electricity, 17.8% for gas for heating, 10.1% for oil, and 1.9% for integrated energy. The nationwide average energy consumption per air conditioning area is 74.4 kWh/m^2^ [3]. Kim et al. investigated changes in the energy sources of school buildings due to changes in educational facility standards. Their study results showed that the power consumption per classroom was 0.06 kWh in the 1970s, but this increased by 8.8 times to 0.53 kWh in the 1980s due to industrial development and changes in environ-mental standards. Owing to changes in environmental and environmental hygiene standards in the 1990s, the power consumption per classroom increased to 2.03 kWh, which was 3.8 times that in the 1980s. It also doubled to 3.99 kWh in the 2000s [4].

Adel Alshibani identified factors that affect energy consumption in school facilities located in eastern Saudi Arabia through artificial neural network analysis. The analysis results showed that the developed neural network model had an accuracy of 87.5%, and the correlation between the school type and air conditioner was found to be highest (0.95) [5]. Kim et al. analyzed the 5-year energy consumption measurement data of nine middle schools in Daegu, Korea by month and year. They found that the energy consumption ranged from 67 to 240 kWh/m^2^/yr and that the energy consumption was significantly affected by the school size according to the population density and population [6].

Branko et al. analyzed the use of simulated building energy based on randomly selected combinations of user-related variables measured at three low-energy schools built in Sweden. The results of the analysis showed that energy performance was 30–160 kWh/m^2^.a as the combination of variables was changed. The study shows that while the energy needed to set indoor air temperature and operate the ventilation system has a broad impact, tenants' electricity use has a slightly lower impact on building energy use [7, 8].

Pereira L.D et al. were intended to achieve functional benchmarking based on the actual operation of the school building by utilizing the results released through intensive literature survey on energy consumption of the school [9]. Christine D. et al. analyzed sensitivity to rank the importance of various factors affecting energy use. It also visited 15 schools across the UK and the purpose of this visit was to collect data on several factors associated with building energy use [10]. Mohamed M. O. et al. analyzed energy consumption for 10 years in samples of 30 school buildings in Manitoba, Canada. The results showed that the median value of total energy consumption in these schools is higher than other Canadian benchmarks. It is emphasized that the results are changed according to the measurement criteria used to report school energy consumption and that these measurement standards need to be standardized [11]. The limitations of domestic and overseas studies related to energy in educational facilities include the impossibility of identifying detailed energy consumption in individual buildings due to the use of statistical data, difficulty in identifying detailed information on energy sources aside from electricity and gas, and the possible unreliability of alternative analyses through simulation in terms of energy consumption. Saving energy is an important activity performed by managers who are most aware of school operating conditions, and it involves realizing energy-saving operations according to the building load (energy consumption) characteristics, use of equipment, and operating conditions.

**Figure 1:**
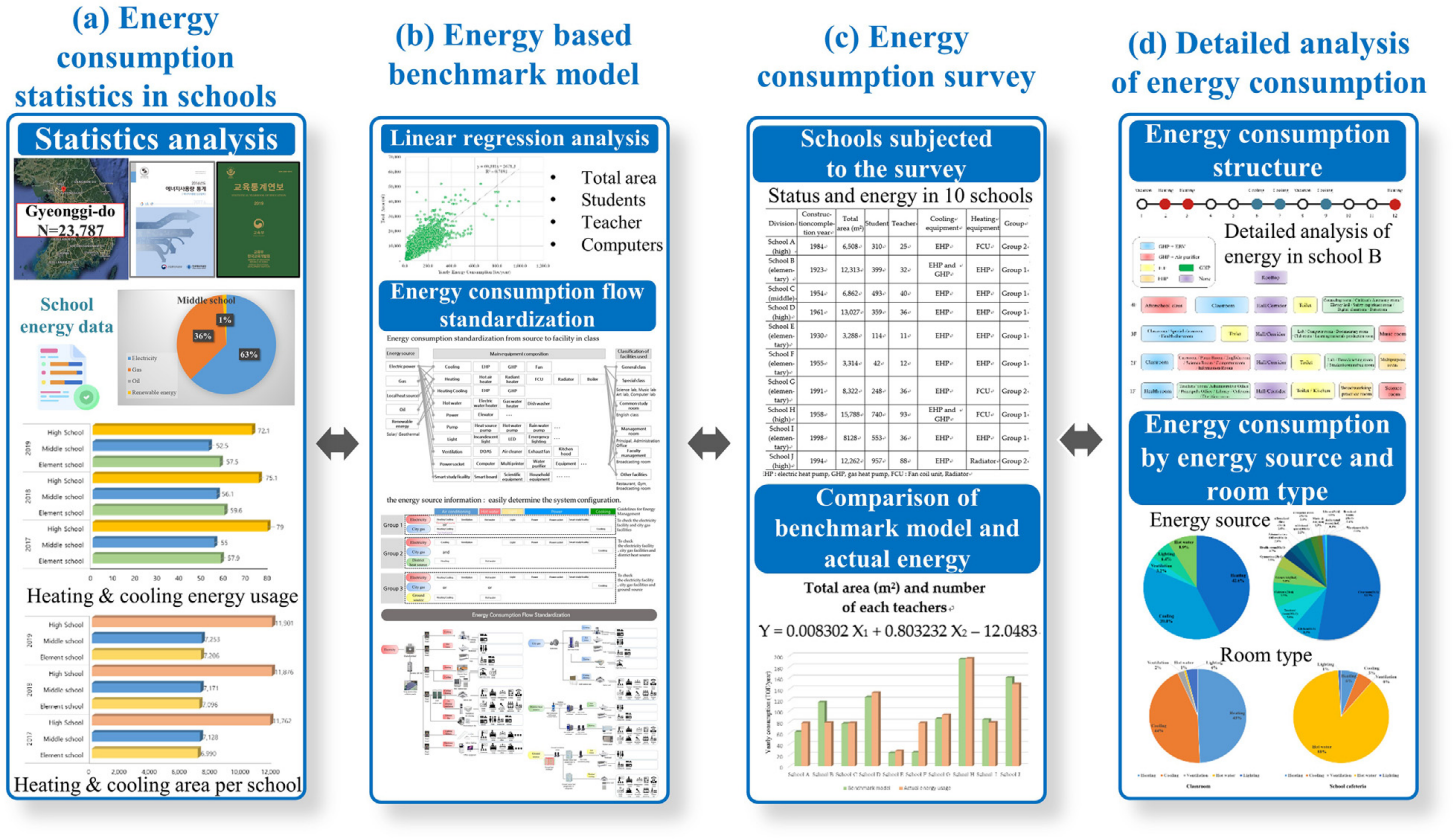
Flowchart of this study ((a) energy consumption statistics in schools, (b) energy based benchmark model, (c) energy consumption survey, (d) detailed analysis of energy consumption).

This study aims to develop the energy based benchmark model (EBBM), statistical data related to energy consumption in schools were analyzed, as shown in Figure 1((a)-(d)). EBBM applicability review is conducted through detailed system analysis of 10 schools. Moreover, energy consumption detail by energy source and room type was analyzed through an in-depth status survey. It can be operated for school facility, through EBBM even non-experts can easily identify energy saving factors. Additionally it helps in understanding the building energy demands in the operating stage of school building diagnosis. This can be also used as a basic survey when energy-saving factors, such as remodeling of school buildings, are applied.

## 2 Energy consumption status in schools

**Figure 2:**
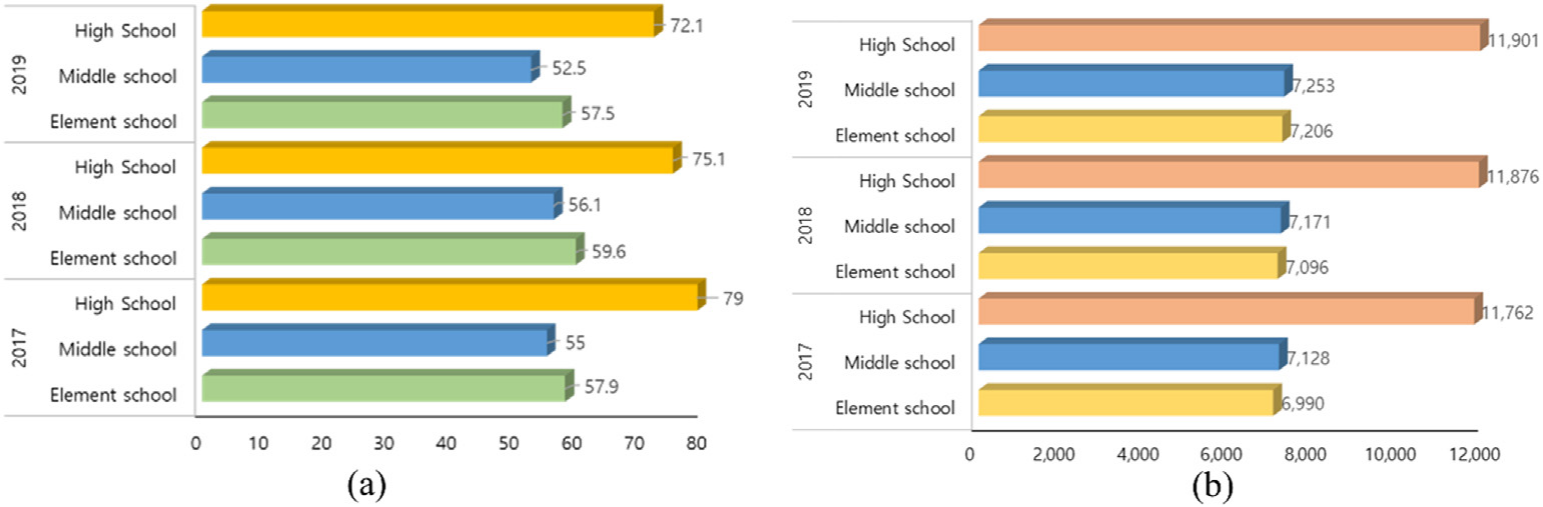
Energy consumption per cooling and heating area of educational facilities ((a) heating cooling energy usage, (b) average heating and cooling area per school).

For schools, energy consumption has continuously increased since 2000 and the year-on-year growth rate is 4.1% on the energy statistics handbook of the Korea Energy Agency [12]. According to the statistical yearbook of education from the Korean Educational Development Institute, the average proportions of electricity, gas, and renewable energy consumption in elementary, middle, and high schools are 67.8%, 30.5%, and 1.7%, respectively [13, 14, 15, 16]. Elementary schools exhibit the highest total energy consumption, followed by high and middle schools. This is because elementary schools have the largest number of students and classrooms and high schools include night classes. For elementary schools, electricity (66.1%) constitutes the largest proportion of energy consumed, followed by gas (31.9%), renewable energy (2.0%), and oil. For middle schools, electricity (68.5%) constitutes the largest proportion of energy consumed, followed by gas (29.6%), renewable energy (1.9%), and oil. For high schools, electricity (68.8%) exhibits the largest proportion of energy consumed, followed by gas (29.9%), renewable energy (1.3%), and oil. From 2017 to 2019, the energy consumption per cooling and heating area was larger in high schools followed by elementary and middle schools, as shown in Figure 2((a)-(b)).

## 3 Energy based benchmark model

**Figure 3:**
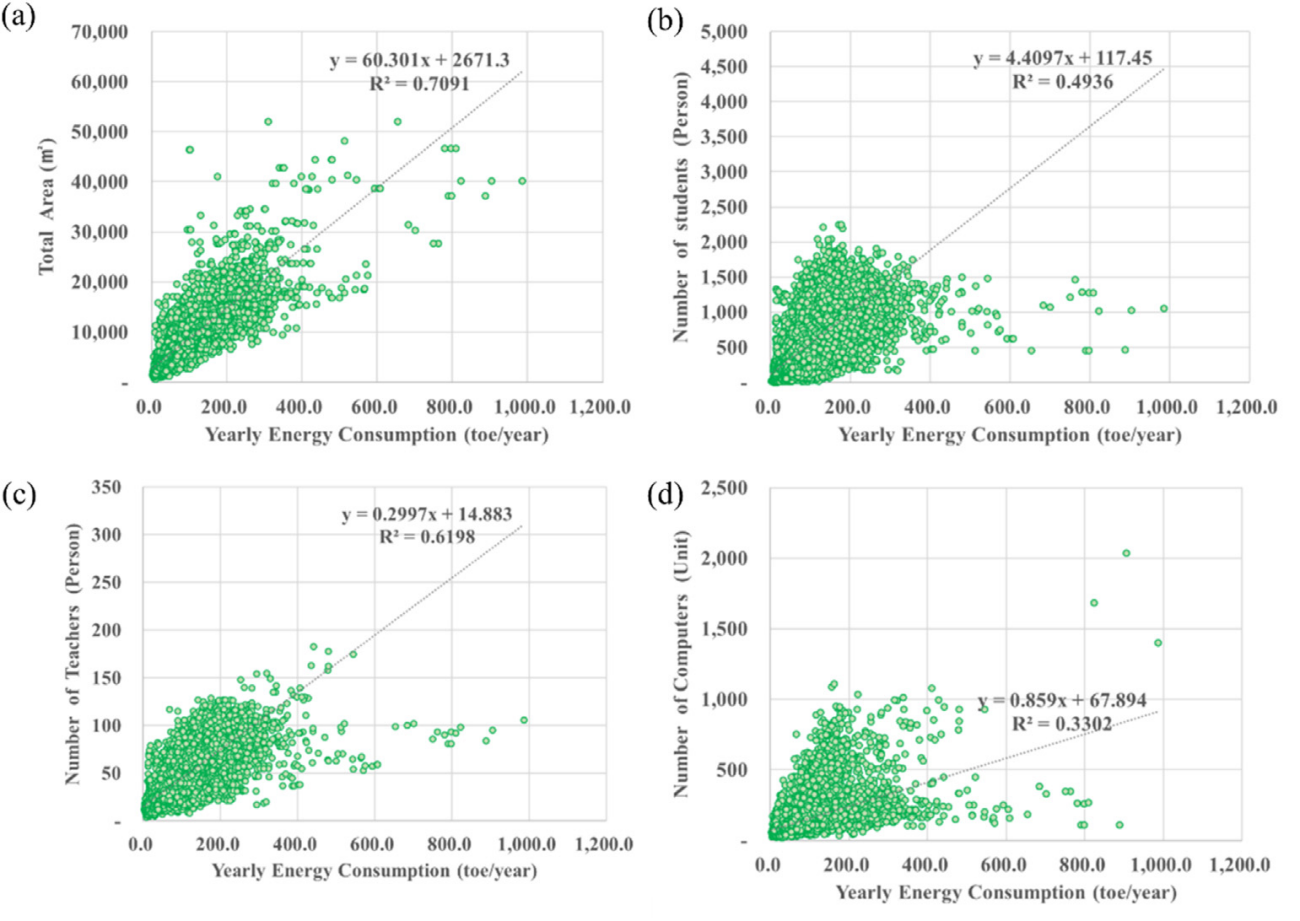
Linear regression analysis of influencing factor ((a) total area, (b) number of students, (c) number of teacher, (d) number of computers (n = 23,787)).

**Figure 4:**
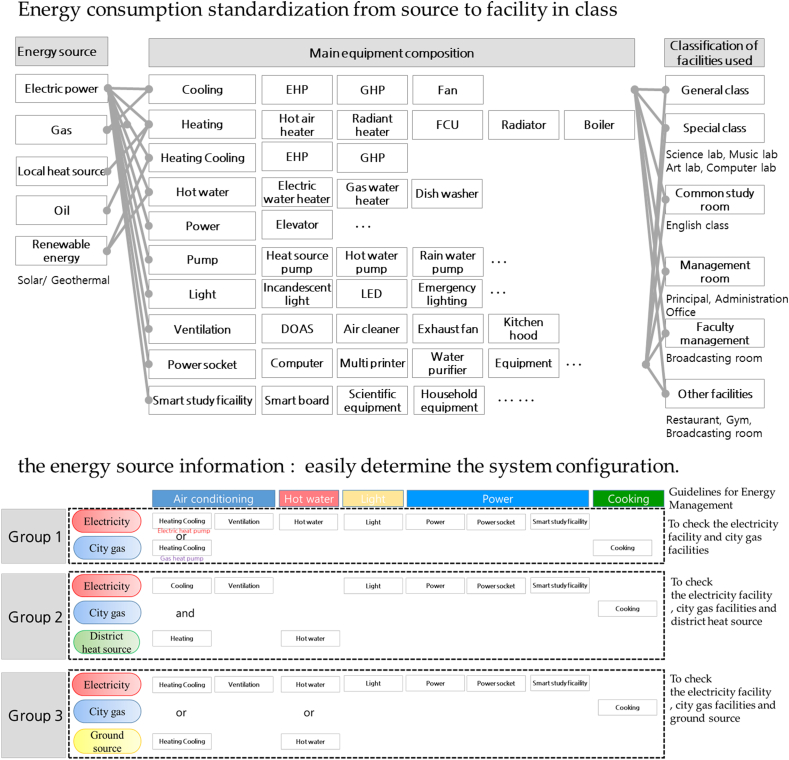
Energy consumption standardization from source (ECFS) to facility in class.

Energy benchmarking analysis is conducted for each influencing factor through Korean school information and energy consumption. After setting each factor according to energy consumption and operating characteristics of elementary, middle and high school in Gyeonggi-do region with the largest number of schools in Korea. A linear regression analysis was performed by setting the number of students, total area, number of teachers, number of computers, and age of building as influencing factors. This correlation analysis reviewed the total energy consumption for 23,787 elementary, middle, and high schools. This analysis may differ in details such as the shape and envelope of the building, but is intended to determine factors in the order of influence through overall trend analysis. Figure 3((a)-(d)) showed the results of linear regression analysis of influencing factor. Table 1 showed R^2^ value of influencing factor based on linear regression analysis. In the model equation of Table 1, Y means annual energy consumption such as electricity and gas, and X means a factor. As a result of a linear regression analysis of the factors for all Gyeonggi region schools, it was found that there was a correlation in the order of total area (0.7091), number of teachers (0.6198), number of students (0.4936) and number of computers (0.3302). It can be seen that the area-related factors and the factors related to the number of people which are related to scale of school have a high influence on the school energy consumption. Before analyzing each factor, the number of students was expected to have a correlation as the most direct influencing factor among the personnel-related factors, but showed a smaller correlation than the number of teachers at 0.4936. Since the number of teachers and the number of students has a high correlation, it is presumed that the factors were influenced by multi collinearity in the regression analysis. The analyzed of multi complex correlation coefficient, the R^2^ value of total area and number of students was 0.8507. The results of the total area and number of each teachers was 0.8609. The results of R^2^ value of total area and number of computers was 0.8435. To analyze the types of educational facilities, domestic school facility plans, design guidelines, and facility standards development research and guidebooks were investigated [17]. In this study also, the classification of facilities was analyzed through the Korean educational facility database of 120,095 schools. As shown Figure 4, there are the energy consumption flow from source to facility in class [18]. This made it possible to standardize the applied system. As a project of the competent education office in each region, design guidelines reflecting the specificity of each school level and regional characteristics, and standards considering the facility sector in accordance with regional education specialization were presented. Through this, space characteristics and facility characteristics of educational were identified. School facilities are classified by room type (Class room, Teaching support room, Management room etc.) and purpose, and it is necessary to analyze the characteristics of energy consumption and energy-saving points by sector (room type).

**Table 1:** R^2^ value of influencing factor based on linear regression analysis.

Factor	Linear regression (R^2^)
	Model equation
Total area (m^2^)	0.7091 Y = 60.301 X + 2671.3
Number of students	0.4936 Y = 4.4097 X + 117.5
Number of each teachers	0.6198 Y = 0.2997 X + 14.9
Number of computers	0.3302 Y = 0.859 X + 67.9
Total area (m^2^) and number of students	0.8507 Y = 0.00992 X_1_ + 0.0285 X_2_ - 6.2613
**Total area (m**^**2**^**) and number of each teachers**	0.8609 Y = 0.008302 X_1_ + 0.803232 X_2_ – 12.0483
Total area (m^2^) and number of computers	0.8435 Y = 0.011185 X_1_ + 0.043271 X_2_ – 8.2011

Calculation of the number of classrooms suit-able for the curriculum, size of classrooms, and facility standards for each school level are presented. And in relation to facilities, the contents of the installation system for each school were analyzed using the internal investigation data of the Ministry of Education. As a result of generalization analysis of spatial types of educational facilities, the following spatial analysis could be generalized. In addition, for educational facilities, the main equipment configuration has been generalized for each energy source, and the configuration of the applied equipment has been generalized according to the classification of each facility used. In this research we will use the standardization definition we developed in an earlier publication [19].

**Figure 5:**
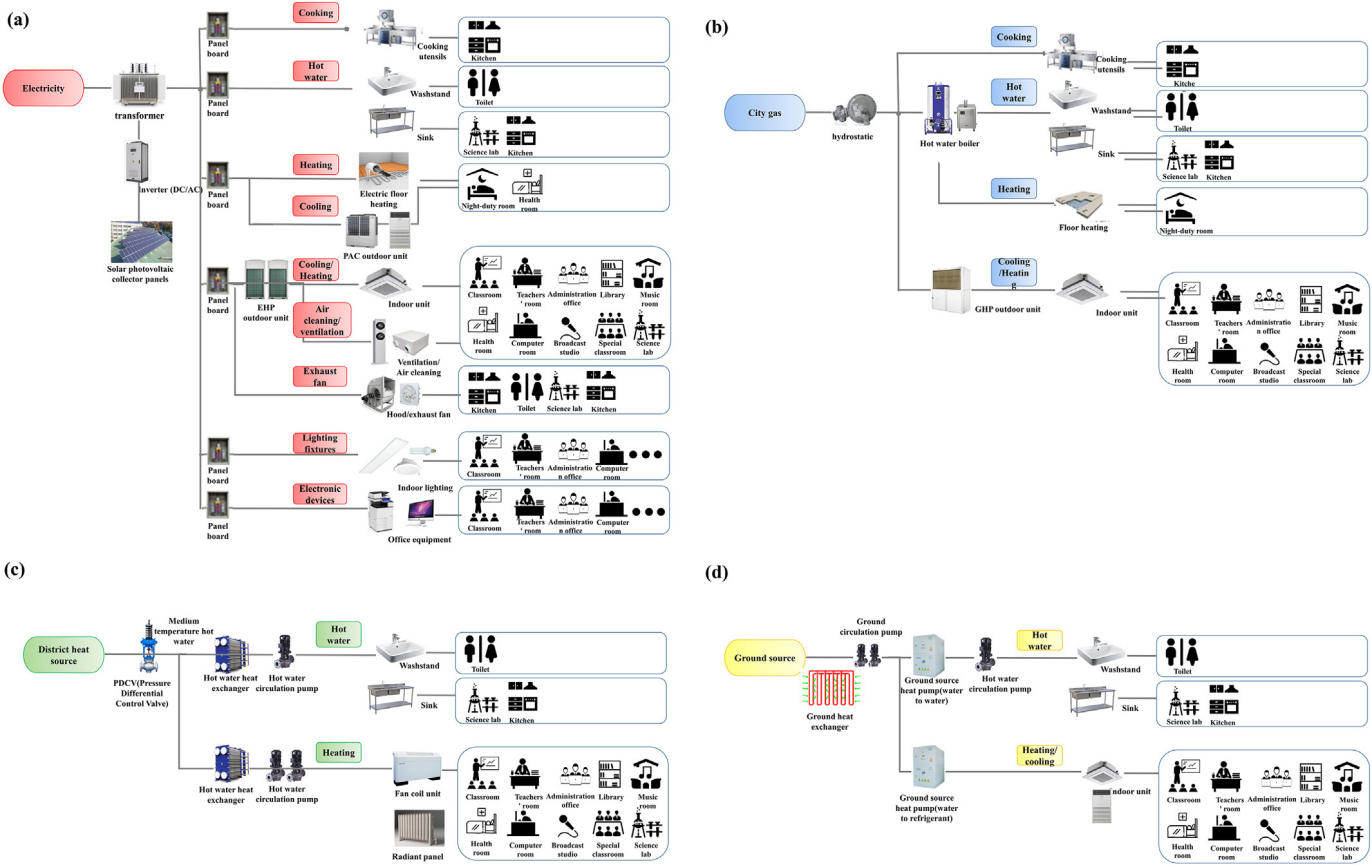
Energy consumption flow standardization in educational facilities ((a) Electric, (b) City gas, (c) District heat source, (d) Ground source).

Simultaneously with the start of the survey of energy consumption, it can be easily understanding the current school electrical system, gas system, local heat source system, oil system and renewable energy. Standard types by energy source in elementary, middle, and high schools were classified as shown in Figure 5((a)-(d)) [18]. The facility structure was similar, with most schools using EHP (Electric Heat Pump), so it was standardized as shown in Figure 5((a)–(d)). For the city gas energy flow, heating, hot water, and cooking heat sources are supplied through a hot water boiler, and cooling and heating are performed using a gas heat pump. For the electricity energy flow, cooling/heating, cooking, hot water, air cleaning/ventilation, exhaust, lighting, and electronic device heat sources are supplied through panel boards from a transformer connected to renewable energy sources. For district heat sources, hot water and heating sources are supplied using primary medium temperature water through a pressure differential control valve, heat exchangers, and circulation pumps. For the ground source energy flow, heating/cooling and hot water heat sources are supplied using heat pumps through the primary ground heat exchanger and circulation pump. In per-forming energy-saving activities, it is important to identify the energy consumption status of the target school. It is also important to identify the reduction target. As above, by using ECFS (city gas, electricity, district heat source, ground source), it is possible to easily understanding the system configuration according to the heat source used in each school. In addition, energy conservation activities are systematized in stages as a target management tool through ECFS that reflect the characteristics of the school. In addition, energy conservation activities are systematized in stages as a target management tool through ECFS that reflect the characteristics of the school.

## 4 Results of field energy consumption survey

Among 120,095 elementary, middle, and high schools nationwide, 10 were selected for this study, taking into account the number of students, construction year, building size, cooling and heating heat sources, and geographic conditions. The target buildings were subjected to an energy survey considering the energy consumption standardization from source to facility for the verification of energy consumption flow standardization. The field energy consumption survey of each school was conducted after obtaining prior consent from the participating schools.

The energy survey was conducted in the following sequence: 1) Identification of the general status of the school facilities, facility operation characteristics, and energy-saving practices through an interview with school officials; 2) acquisition of data on construction, equipment, and energy consumption through the survey form; 3) on-site survey of major facilities, such as classrooms, administration offices, and computer rooms; 4) collection of documents on mechanical and electrical equipment. Here, the detailed analysis of buildings where electrical and gas heat pumps, which are representative of cooling and heating heat sources, was undertaken for the target buildings as the energy survey. As shown in Table 2, the 10 facilities assessed are located in Gyeonggi-do, Gangwon-do, and Seoul, and assessment took place from September to November 2020. The school buildings were di-vided based on geographic conditions, age, and cooling and heating energy sources to select survey targets. Most of the schools used electricity and city gas in group 1 (Figure 4). For the 10 target schools to be surveyed, data on gas, electricity, and district heat sources as well as energy consumption in past years were collected, and data from 2017 to 2019 were analyzed after removing missing data and outliers. The 3-year average use of gas, electricity, and district heat sources as well as their petroleum conversion tons were determined.

**Table 2:** Summary of the buildings subjected to the survey.

Division	Constructioncompletion year	Total area (m^2^)	Student	Teacher	Cooling equipment	Heating equipment	Group
School A (high)	1984	6,508	310	25	EHP	FCU	Group 2
School B (elementary)	1923	12,313	399	32	EHP and GHP	EHP	Group 1
School C (middle)	1954	6,862	493	40	EHP	EHP	Group 1
School D (high)	1961	13,027	359	36	EHP	EHP	Group 1
School E (elementary)	1930	3,288	114	11	EHP	EHP	Group 1
School F (elementary)	1955	3,314	42	12	EHP	EHP	Group 1
School G (elementary)	1991	8,322	248	36	EHP	FCU	Group 2
School H (high)	1958	15,788	740	93	EHP and GHP	FCU	Group 1
School I (elementary)	1998	8128	553	36	EHP	EHP	Group 1
School J (high)	1994	12,262	957	88	EHP	Radiator	Group 2

EHP: electric heat pump, GHP, gas heat pump, FCU: Fan coil unit, Radiator.

**Figure 6:**
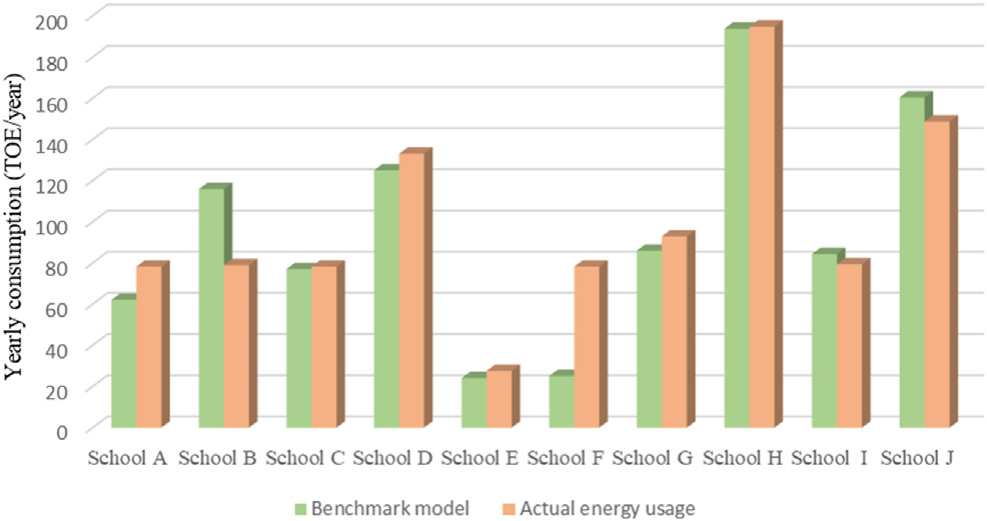
Comparison of benchmark model and real data energy consumption.

The petroleum conversion ton was introduced to compare the amounts of each energy source because they have different masses and volumes. The heat generated from one ton of petroleum was standardized based on the calories (kcal), and the unit of tons of oil equivalent (TOE) was used. Table 3 shows the real annual energy source consumption status and the annual energy consumption by total school area. It also showed the results of difference between calculated value by the benchmark model and actual yearly consumption. Except for schools A and B, the difference between the calculated value and the actual value was confirmed to be within 10%. Through the benchmarking model, it is possible to compare energy consumption for buildings of different sizes. Figure 6 showed the calculated benchmark model value and the real energy consumption. In addition, the representativeness of the 10 schools was achieved through the benchmark model shown in Figure 6 and the verification of the energy usage error rate of the actual energy usage.

**Table 3:** Compared to real annual energy consumption and benchmark model calculated results.

Division	Gas	Electricity	District heating	Actual yearly consumption	TOE per area	Bench mark model calculated	Difference between calculated value and real value
	Nm^3^	kWh	Mcal	TOE	(TOE/m^2^)	TOE	%
School A	11,571	240,306	112,967	78.23	0.0120	62.06	79
School B	21,756	246,965	-	78.94	0.0064	115.88	147
School C	11,856	321,703	-	78.23	0.0114	77.05	98
School D	13,430	520,939	-	133.10	0.0102	125.02	94
School E	-	120,524	-	27.60	0.0084	24.08	87
School F	-	118,952	-	22.90	0.0069	25.10	110
School G	8,769	288,253	17,890	92.92	0.0112	85.96	93
School H	756,411	20,920		194.75	0.0123	193.72	99
School I	19,750	257,807		79.36	0.0098	84.35	106
School J	20,080	522,561	8,316	148.7	0.0121	160.44	108

TOE, tons of oil equivalent.

School H showed the highest energy consumption by combination heating and cooling equipment. The high schools (School A, D, H, J) tended to exhibit the highest power consumption, followed by middle and elementary schools. This appears to be because the high schools have a long class time and use energy for longer due to night classes. In addition, the middle schools consumed more power than the elementary schools. This appears to be because the number of students per classroom was larger for the middle schools compared to the elementary schools, and the average class length was also longer for middle schools. When the annual energy consumption (TOE/year) and total floor area were reviewed, it was found that the energy consumption tended to be higher as the school size increased. This appears to be due to an increase in the number of cooling/heating and lighting devices as well as in the number of students as the school size increased to a total floor area. The proportional relationship between the total floor area of a building and its energy consumption has also been revealed in many previous studies.

## 5 Detailed analysis of energy consumption by energy source and room type

### 5.1 School status and energy consumption structure

**Figure 7:**
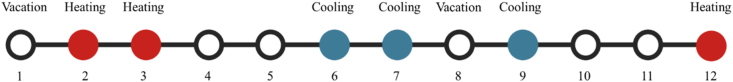
School heating and cooling periods.

**Figure 8:**
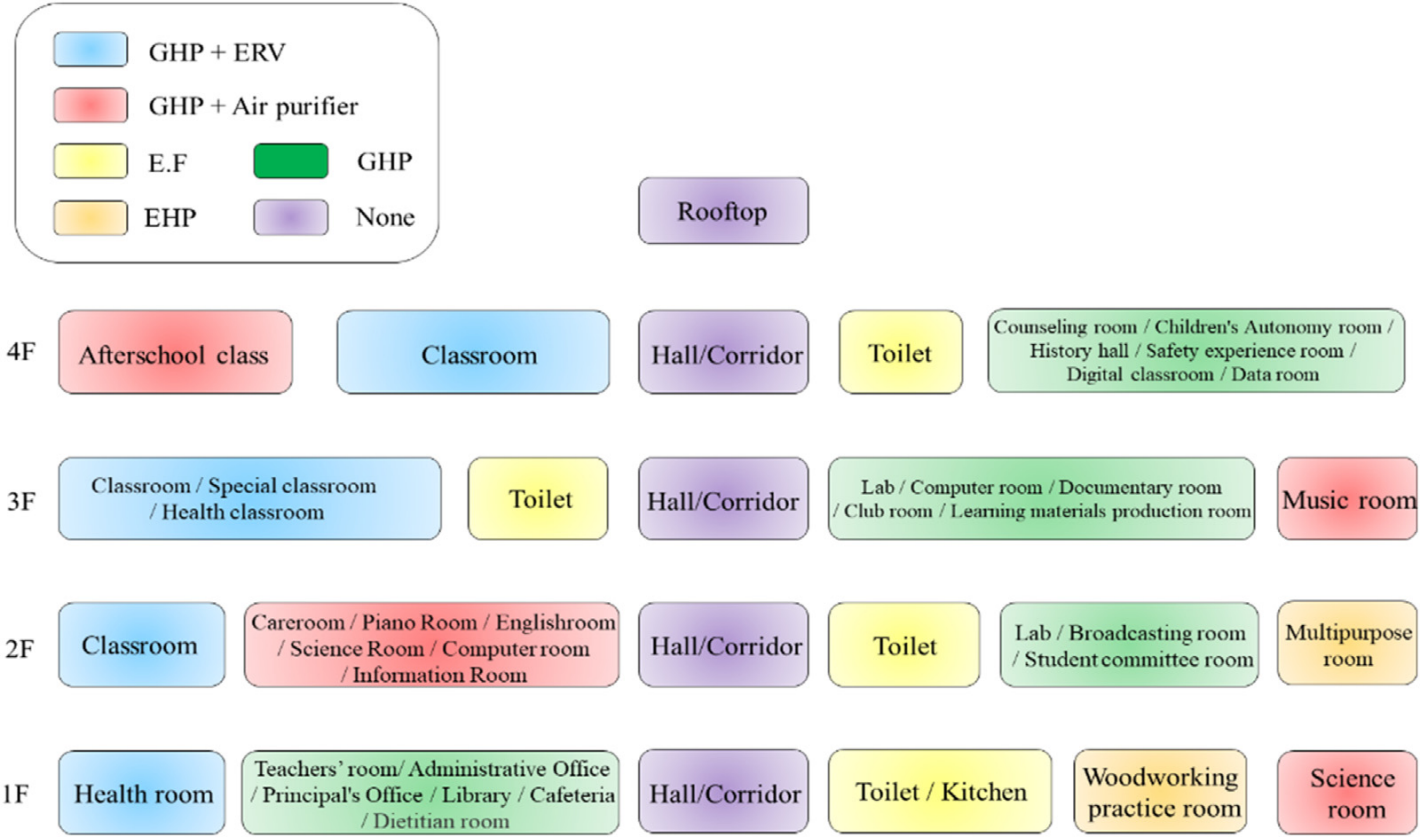
The heating, ventilation and air conditioning (HVAC) system of elementary School B.

Table 4 provides information on the school B subjected to the status survey. It is a four-story building with a building area of 3,078 m^2^ and a total floor area of 12,313 m^2^. As of 2019, 399 students in 71 classrooms, including the first, second, and third grade as well as special and learning classrooms, attended this school with approximately 32 educational personnel. Table 5 shows the operating hours for each room as of 2019. These data were used as basic data to derive the energy consumption by school use and room. The number of school days per year is approximately 210 days, excluding vacations, but heating and cooling periods were derived as shown in Figure 7. After obtaining the architectural documents of School B, the indoor space was separated by use and the area was calculated. Figure 8 shows the status of mechanical equipment in the elementary School J with ECFS-city gas and ECFS-electricity. A gas heat pump, which uses city gas as fuel (ECFS-city gas). In addition, hot water was supplied to the kitchen and toilets using a gas hot water boiler and the central supply method (ECFS-city gas).

**Table 4:** Outline of the survey school.

Category	Information	Image
Location	Gyeonggi-do Paju	
Total area	12,313 m^2^	
Building area	3,078 m^2^	
Type	Elementary school	
Floors	1–4 F

**Table 5:** School daily operating hours.

Room name	Daily operating hours	Room name	Daily operating hours
Classroom	9.0	Teachers' room	10.0
Broadcast studio	1.0	Warehouse	1.0
Chemical room	5.0	Audiovisual room	1.0
Science lab	5.0	Music room	3.0
Kitchen	8.0	Art room	3.0
School cafeteria	3.0	Computer room	5.0
Night-duty room	16.0	Health room	10.0
Afterschool classroom	1.5	Library	3.0

Regarding the status of the electrical equipment (ECFS-electricity), electricity-driven heat pumps were applied in some rooms, such as the woodworking practice room, multipurpose room, and gymnasium. In addition, electricity was supplied to the heat recovery ventilation system, air purifiers, lighting, and indoor devices.

### 5.2 Energy consumption by energy source and room type

To examine the proportion of energy consumption by use and room type in School B, the actual energy consumption and use patterns in previous years were analyzed. The actual electricity and gas consumption patterns from 2017 to 2019 were analyzed, and the results were used as basic data for examining the energy consumption per unit area.

**Figure 9:**
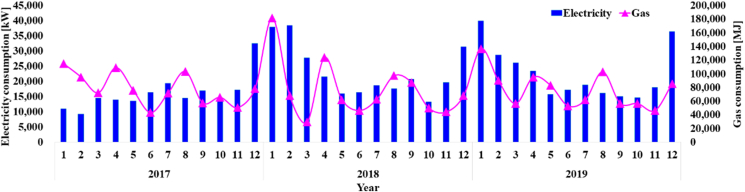
Three-year energy consumption.

Figure 9 shows the electricity and the gas energy use pattern in past years. It was found that the gas consumption tended to decrease during the spring vacation, in February and March for enrollment and graduation, and in May and June when seasons changed, but increased in June to August when cooling was performed and December and January when heating was performed. For the building, the proportion of gas energy use was expected to be larger because gas heat pumps, which are used as cooling and heating equipment, are used, but it was found that gas accounted for 28.4% (0.0041 TOE/year) of the total energy consumption (0.0143 TOE/year) and electricity for 71.6% (0.0103 TOE/year).

**Figure 10:**
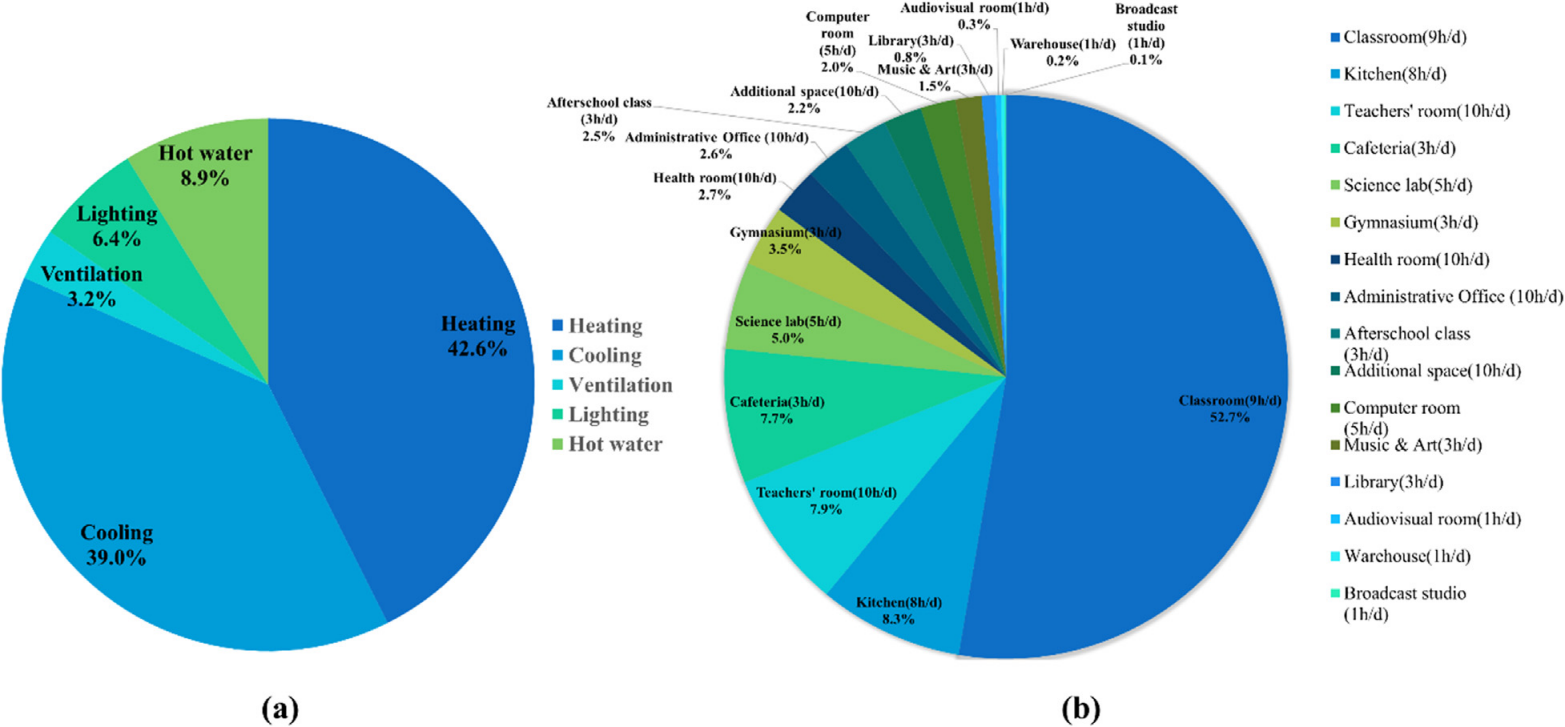
Energy consumption ratio by energy source and room ((a) energy source, (b) room type).

**Figure 11:**
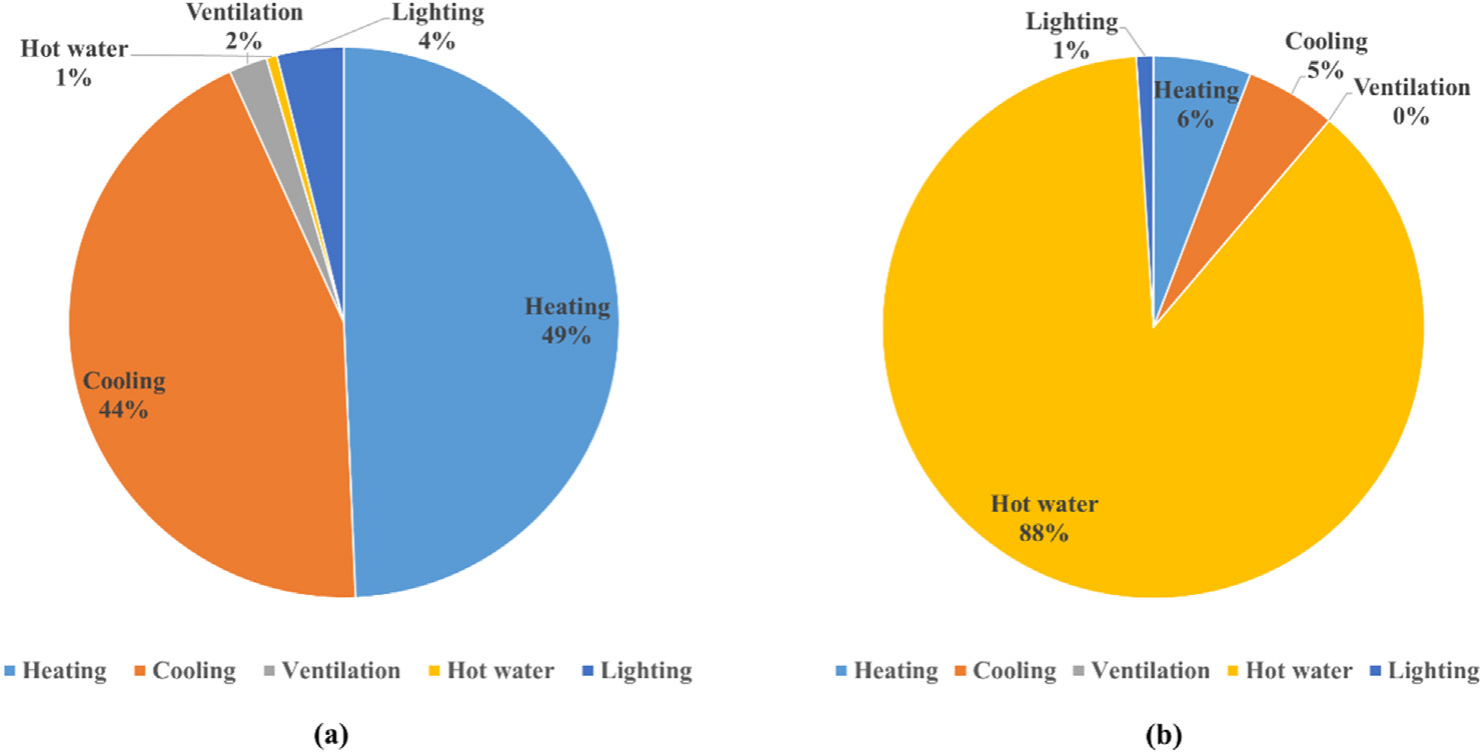
Energy consumption in the classroom and school cafeteria ((a) classroom, (b) school cafeteria).

In Figure 10((a)-(b)), the energy consumption during the vacation periods is estimated to be the base load of the school building because cooling and heating are not performed during these periods. With regards to the distribution of energy consumption by use, it can be mainly divided into electricity energy for lighting, general devices, and ventilation and gas energy for cooling/heating equipment and hot water supply. As examined above, the total energy consumption of the reference school was 78.94 TOE/year, and heating energy constituted the largest proportion at 42.6%, followed by cooling (39.0%), hot water supply (8.9%), lighting (6.4%), and ventilation (3.2%), as shown in Figure 11((a)-(b)).

The proportion of ventilation energy was small because the ventilator power and the number of ventilators installed were relatively smaller compared to those of other equipment. For the school, except for the cooling and heating equipment that represent a considerable proportion of energy consumption, gas energy for cooking in the kitchen and the central hot water supply in the school accounted for 8.9%. Based on the above analysis results, it can be concluded that it is first necessary to introduce technology for reducing cooling and heating gas energy use (ECFS-city gas) and to convert the central hot water supply method with heat loss and low load responsiveness in winter to an energy-saving method when element technologies to reduce energy consumption are applied to schools that use gas heat pumps. It is necessary to examine measures that can minimize energy loss, such as the application of electric water heaters with no heat transfer loss, supplying cooling and heating energy using photovoltaic systems.

Classrooms consumed the largest amount of energy in School B and the use of cooling and heating energy constituted the largest proportion, as shown in Figure 11. Since the cafeteria uses a large amount of hot water during cooking, the use of hot water energy constituted the largest proportion, followed by heating, cooling, and lighting. Unlike other schools, the cafeteria uses a lot of gas energy, so gas-related variables that cannot be reflected in the benchmarking model were identified. Therefore, it is analyzed that the result value of the benchmarking model has the largest difference from the actual value.

## 6 Conclusion

The degradation of building performance (e.g., low-airtightness, low-insulation, and condensation) due to the aging of school buildings has increased the energy consuming. The need for energy management measures for school buildings is increasing. Saving energy is an important activity performed by managers who are most aware of school operating conditions, and it involves realizing energy-saving operations according to the building load (energy consumption) characteristics, use of equipment, and operating conditions.

(1) In this study, to develop the energy based benchmark model (EBBM), statistical data related to energy consumption in schools were analyzed. EBBM applicability review is conducted through detailed system analysis of 10 schools. Moreover, the data related to energy consumption in schools were analyzed.
(2) To derive a bench-mark model, the relationship between school use variables, total floor area, number of students, teachers, and computers, and energy use was analyzed. As a result of a linear regression analysis of the factors for all Gyeonggi region schools (n = 23,787), it was found that there was a correlation in the order of total area (R^2^ = 0.7091), number of teachers (R^2^ = 0.6198), number of students (R^2^ = 0.4936) and number of computers (R^2^ = 0.3302). Energy consumption had the highest correlation with the order of total area and number of teachers (R^2^ = 0.8609).
(3) The status survey, the ECFS types of the 10 schools were confirmed that it matches well with the ECFS type. Most of the schools used electricity and city gas in group 1.The use of the energy consumption flow standardization of school facilities that even non-experts can easily identify energy saving factors. It also helps in understanding the building energy demands in the operating stage of school building diagnosis. The difference between the bench mark model calculated value and the actual value was confirmed to be within 10%. Through the benchmarking model, it is possible to compare energy consumption for buildings of different sizes.
(4) As a results of analyzing the energy structure of School B that the benchmarking model has the lowest accuracy. For the school B, except for the cooling and heating equipment that represent a considerable proportion of energy consumption, gas energy for cooking in the kitchen and the central hot water supply in the school accounted for 8.9%. it was found that gas accounted for 28.4% of the total energy consumption and electricity for 71.6%. A large amount energy consumption could be saved if the ECFS-electricity and ECFS-city gas facility could be changed to high efficient facilities and to operate efficiently.
(5) As future work, it will be necessary to develop an energy saving operation guideline that can perform effective energy saving activities of the school facilities. It would be important to make a display platform for the chief energy manger (teachers), student to share the energy consumption. For that reason, this re-search is the necessary to step to start with the understand school facilities and implement energy management in Korea.

## Declarations

### Author contribution statement

Beungyong Park, Byeong-Un Kang, Doo-Yong Park: Conceived and designed the experiments; Performed the experiments; Analyzed and interpreted the data; Contributed reagents, materials, analysis tools or data; Wrote the paper.

### Funding statement

This work was supported by the 10.13039/501100003725National Research Foundation of Korea grant funded by the Korea government (MSIT, MOE) and (NRF-2019M3E7A1113092).

### Data availability statement

Data included in article/supp. material/referenced in article.

### Declaration of interests statement

The authors declare no conflict of interest.

### Additional information

No additional information is available for this paper.
